# Efficacy and safety of remimazolam versus propofol for intraoperative sedation during regional anesthesia: A phase II, multicenter, randomized, active‐controlled, single‐blind clinical trial

**DOI:** 10.1002/ibra.12163

**Published:** 2024-06-16

**Authors:** Ting‐Ting Li, Lu Yin, Yue‐Xin Huang, Xiu‐Hong Wang, Yan‐Huan Wei, Yong Wang, Shi‐Wei Yang, Genoveva B. da Graca Cunha, Fei Liu

**Affiliations:** ^1^ Department of Anesthesiology West China Hospital, Sichuan University Chengdu Sichuan China; ^2^ West China School of Nursing Sichuan University Chengdu Sichuan China; ^3^ Graduate School of Education Beijing Foreign Studies University Beijing China; ^4^ Division of Gastrointestinal Surgery, Department of General Surgery West China Hospital, Sichuan University Chengdu Sichuan China; ^5^ Hospital Ayres Menezes Sao Tome Sao Tome and Principe

**Keywords:** HR7056, propofol, regional anesthesia, remimazolam, sedation

## Abstract

This study aimed to evaluate the efficacy and safety of remimazolam for intraoperative sedation during regional anesthesia. It was a phase II‐multicenter, randomized, single‐blind, parallel‐group, active‐controlled clinical trial (No. ChiCTR2100054956). From May 6, 2021 to July 4, 2021, patients were randomly enrolled from 17 hospitals in China. A total of 105 patients aged 18–65 years who underwent selective surgery under regional anesthesia were included. Patients received different sedatives with different dosages: 0.1 mg/kg remimazolam (HR), 0.05 mg/kg remimazolam (LR), or 1.0 mg/kg propofol (P) group, followed by a maintenance infusion. Main outcome measures included the efficacy of sedation measured by Modified Observer's Assessment of Alertness/Sedation Scale (MOAA/S) levels (1–4, 1–3, 2–3, 3, and 2–4) during the sedation procedure (the duration percentage) and incidence of adverse reactions. It showed that the duration percentage of MOAA/S levels 1–4 was 100.0 [8.1]% (median [interquartile range]), 89.9 [20.2]%, 100.0 [7.7]% in the HR, LR, and P groups, respectively. The percentage of patients in the HR, LR, and P groups who achieved MOAA/S levels 1–4 within 3 min after administration was 85.7%, 58.8%, and 82.9%, respectively. However, the time to recovery from anesthesia after withdrawal of sedatives (7.9 ± 5.7 min), incidence of anterograde amnesia (75%), and adverse effects were not statistically significant among the three groups. These findings suggest that a loading dose of remimazolam 0.1 mg/kg followed by a maintenance infusion of 0–3 mg/kg/h provides adequate sedation for patients under regional anesthesia without increasing adverse reactions.

## 1 INTRODUCTION

Patients remain conscious during local anesthesia, which may increase perioperative anxiety^1^ that exposes patients to greater surgical risk,^2^ such as hemodynamic fluctuations, pain, and so forth.^3^ Regional anesthesia‐assisted sedation has the advantages of reducing intraoperative bleeding, fast postoperative recovery, and so on, making it conducive to clinical application.^4^ However, at present, commonly used clinical sedative drugs include propofol, midazolam, dexmedetomidine, and so on, each with limitations that restrict their utility. Propofol may induce body moving, severe respiratory circulation inhibition, necessitating close monitoring by anesthesiologists.^5^, ^6^ Midazolam tends to accumulate drugs after repeated use and is susceptible to interact with multiple drugs.^7^ Dexmedetomidine is a highly selective α_2_ adrenergic receptor agonist, which may cause severe bradycardia.^8^ Therefore, it is of great significance to develop more ideal sedative drugs for safe clinical use.

Remimazolam, a short‐actingγ‐aminobutyric acid A (GABAa) receptor agonist, is a new benzodiazepine developed to provide sedative effects with a short half‐life. It has the advantage of rapid onset/failure, stable sedation, short recovery time, low potential for drug interactions, and reversible agents.^9^, ^10^ Unlike midazolam and propofol, remimazolam undergoes organ‐independent metabolism to form an inactive metabolite. Furthermore, because remimazolam follows first‐order pharmacokinetics, prolonged infusions or higher doses are unlikely to result in accumulation and extended effects.^11^ However, there are no relevant studies for its sedation during regional anesthesia. Remimazolam tosilate (HR7056), belongs to “a new indication preparation containing known active ingredients.” To date, many clinical trials for procedural sedation have been completed and have reported that remimazolam has satisfactory sedative and hypnotic effect.^12^, ^13^ Therefore, in this study, different concentrations of remimazolam were compared with propofol (active control, the most commonly used sedative in clinical^4^) to analyze the safety and effectiveness of remimazolam for sedation during regional anesthesia and to explore the dose range.

## 2 MATERIALS AND METHODS

### 2.1 Ethics and registration

This study was conducted in accordance with Good Clinical Practice Guidelines and the principles of the Declaration of Helsinki, approved by the local ethics committee (Ethics Committee on Clinical Trial, West China Hospital of Sichuan University, No. HX‐IRB‐AF‐14‐V4.0, Clinical trial [Western medicine] Review No. 35, 2021. Chairperson Prof Ye‐Rong Yu), and Clinical trial by State Drug Administration (No. CXHL2000187). This study has been registered with the Chinese Clinical Trial Registry (No. ChiCTR2100054956). Written informed consent was obtained from all subjects, a legal surrogate, and the parents or legal guardians for minor subjects, or the requirement for written informed consent was waived by the Institutional Review Board (IRB). All the information of CONSORT 2010 checklist was followed in this study.

### 2.2 Study design

This was a multicenter, randomized, single‐blind, parallel‐group, active controlled clinical trial. The study was conducted in 17 hospitals throughout China from May 6, 2021 to July 4, 2021 (Supporting Information S1).

### 2.3 Patients

A total of 108 patients were enrolled and randomly divided into three groups at a ratio of 1:1:1. High‐dosage remimazolam (HR) groups were subject to 0.1 mg/kg remimazolam (Jiangsu Hengrui Pharmaceutical Co., Ltd., No. 210124AK, 36 mg [by free base]), while low‐dosage remimazolam (LR) groups underwent 0.05 mg/kg remimazolam. As for propofol (P) group, patients were subject to 1.0 mg/kg propofol (AstraZeneca Pharmaceutical Co., Ltd., No. RM031, 50 mL:500 mg).

#### 2.3.1 Inclusion criteria

Eligible patients met the following inclusion criteria: (1) aged 18–65 years; (2) 18–28 kg/m^2^ of body mass index (BMI); (3) American Society of Anesthesiologists (ASA) class I–II; (4) elective surgery (time >30 min) under regional anesthesia in the operation room; and (5) voluntary participation and signed Inform consent form.

#### 2.3.2 Exclusion criteria

Patients with following conditions were excluded: (1) interventional operation; (2) difficulty in respiratory management; (3) psychiatric disorders, cognitive dysfunction, or severe systemic diseases; (4) general anesthesia within 7 days before randomization; and (5) allergy or contraindication to benzodiazepines, propofol, fentanyl, and other pharmaceutical ingredients.

### 2.4 Randomization and blinding

The randomization process was completed using SAS, version 9.4 (SAS, Inc.), and the Interactive Network Response Randomization System (IWRS). The study adopted a single‐blind design, with only subjects being blinded due to the difference in drugs, dosage, and infusion rates during sedation.

### 2.5 Perioperative period

Standard vital signs monitoring (blood pressure, heart rate, respiration rate, blood oxygen saturation [SpO_2_], and oxygenation [oxygen flow rate: 1–2 L/min]) were continuously performed for all patients in the operation room. Following the administration of regional anesthesia and confirmation of its satisfactory effect, baseline Modified Observer's Assessment of Alertness/Sedation Scale (MOAA/S) was evaluated (the MOAA/S level ranges from 0 to 5, with a lower score representing a deeper level of sedation), and sedation procedure was performed successively.

The sedation procedure commenced with the intravenous administration of the study drug and continued until withdrawal. According to the randomization, the load dose of the study drug was injected intravenously (iv), followed by maintenance dose (LR: 0.05 mg/kg body weight [BW] + 0.5 mg/kg/h; HR: 0.1 mg/kg BW + 0.5 mg/kg/h; P: 1.0 mg/kg BW + 3 mg/kg/h). The maintenance dose of remimazolam was adjusted from 0 to 3 mg/kg/h, while that of propofol was adjusted from 0 to 4.5 mg/kg/h. Infusion of drug was stopped immediately (±2 min) after the completion of surgery. The targeted sedation level was maintained at MOAA/S level 1–4, with adjustments made to the infusion rate during the surgery. If the MOAA/S of the patient was still level 5 after the maximum infusion rate for 5 min, rescue sedative medication would be given. Upon achieving the target sedation criteria, patients received intravenous fentanyl (50 mcg) before undergoing surgery. Additional fentanyl (50 mcg) was administered intravenously during the operation if the patient's behavior pain scale (BPS^14^) > 6. The initial intravenous administration was 0.5 mg/kg/h in the HR and LR groups and 3 mg/kg/h in the P group. According to the sedation state, remimazolam was adjusted in a range of 0.2–0.5 mg/kg/h each time, while propofol is adjusted at a range of 0.4–2 mg/kg/h each time. After the operation, the patients were continually monitored until the Aldrete score was^15^ ≥9. Postoperative follow‐ups were conducted on the day following surgery.

During the study, various indicators were assessed, including the type of surgery, duration of the operation, type of regional anesthesia, the vital signs, MOAA/S level, rescue sedation, additional analgesic, anterograde amnesia (defined as the inability of the patient to correctly select a previously seen picture from a set of similar pictures), and any adverse events. Adverse events encompassed injection pain, spontaneous movement during surgery, hypertension (defined as systolic blood pressure [SBP] > 140 mmHg or diastolic blood pressure [DBP] > 90 mmHg), hypotension (defined as SBP < 90 mmHg), bradycardia (defined as heart rate <50 bpm) or tachycardia (defined as heart rate >100 bpm), oxygen saturation (SpO_2_) <90%, postoperative nausea and vomiting (PONV), postoperative delirium (assessed using the Nursing Delirium Screening Score, Nu‐DESC), and any other relevant events. Additionally, both patient and anesthesiologist satisfaction levels were evaluated using a scale ranging from 0 to 10, where 0 indicated dissatisfaction and 10 indicated utmost satisfaction.

### 2.6 Outcome assessments

Primary outcome included the percentage of time (min) maintaining MOAA/S levels 1–4 throughout sedation (duration percentage of MOAA/S levels 1–4).

Secondary outcomes included the percentage of duration of MOAA/S levels 1–3, levels 2–3, level 3, and levels 2–4; percentage of target sedation achieved within 3 min; incidence of rescue sedation; incidence of anterograde amnesia; time from end of sedation procedure to first achieving MOAA/S level 5 and Aldrete ≧9 after operation; sedation satisfaction of anesthesiologist and patients; and incidence and severity of various adverse events during the study.

### 2.7 Statistical analysis

According to data from phase Ib clinical trials, continuous intravenous infusion of remimazolam resulted in good sedation in approximately 80% of patients. Propofol has been used clinically for many years, and almost, all patients can achieve sedation under the existing dosage. Sample sizes of 30 in each group achieve 80% power to detect a noninferiority margin ratio in the group proportions of 1. The test statistic used is the one‐sided score test (Miettinen & Nurminen). The significance level of the test was targeted at 0.0250. Considering the 20% loss of the follow‐up rate, the final plan is to include 36 patients each group.

Statistical analyses were performed using SPSS 25.0 statistical software. Outcome assessments were divided into count data and measurement data. Count data were analyzed by the *χ* ^2^ test or Fisher's exact probability method and expressed as frequency and percentage. If the measurement data were normally distributed, analysis of variance (ANOVA) and Bonferroni were used (expressed as mean ± standard deviation [Std]). Otherwise, the Kruskal–Wallis test would be chosen (expressed as median [range interquartile [IQR]]). Based on the results of the preliminary analysis, duration percentage of MOAA/S levels at 0–1, 1–2, 2–3, 3–4, and 4–5 (adjacent MOAA/S levels) was further analyzed. Spearman correlation analysis was used to analyze the correlation between grouping (HR, LR, and P group), sedation satisfaction of anesthesiologist and patients, and adjacent MOAA/S levels. The results were expressed as correlation coefficients (*r*). Based on the above analysis, patients were divided into two groups according to whether the duration percentage of MOAA/S levels 1–3 was more than 75% (>75%: longer duration of appropriate sedation; ≦75%: shorter duration of appropriate sedation). The binary logistic regression analysis was performed. The results were expressed by odds ratio (OR) value and 95% confidence interval (95% CI).

Statistical significance was accepted at *p* ≤ 0.05 (two‐sided tests). If *χ* ^2^ test, Fisher's exact, or rank sum test were used, the difference was considered statistically significant only when *p* < 0.017 for pairwise comparison was used.

## 3 RESULTS

**Figure 1:**
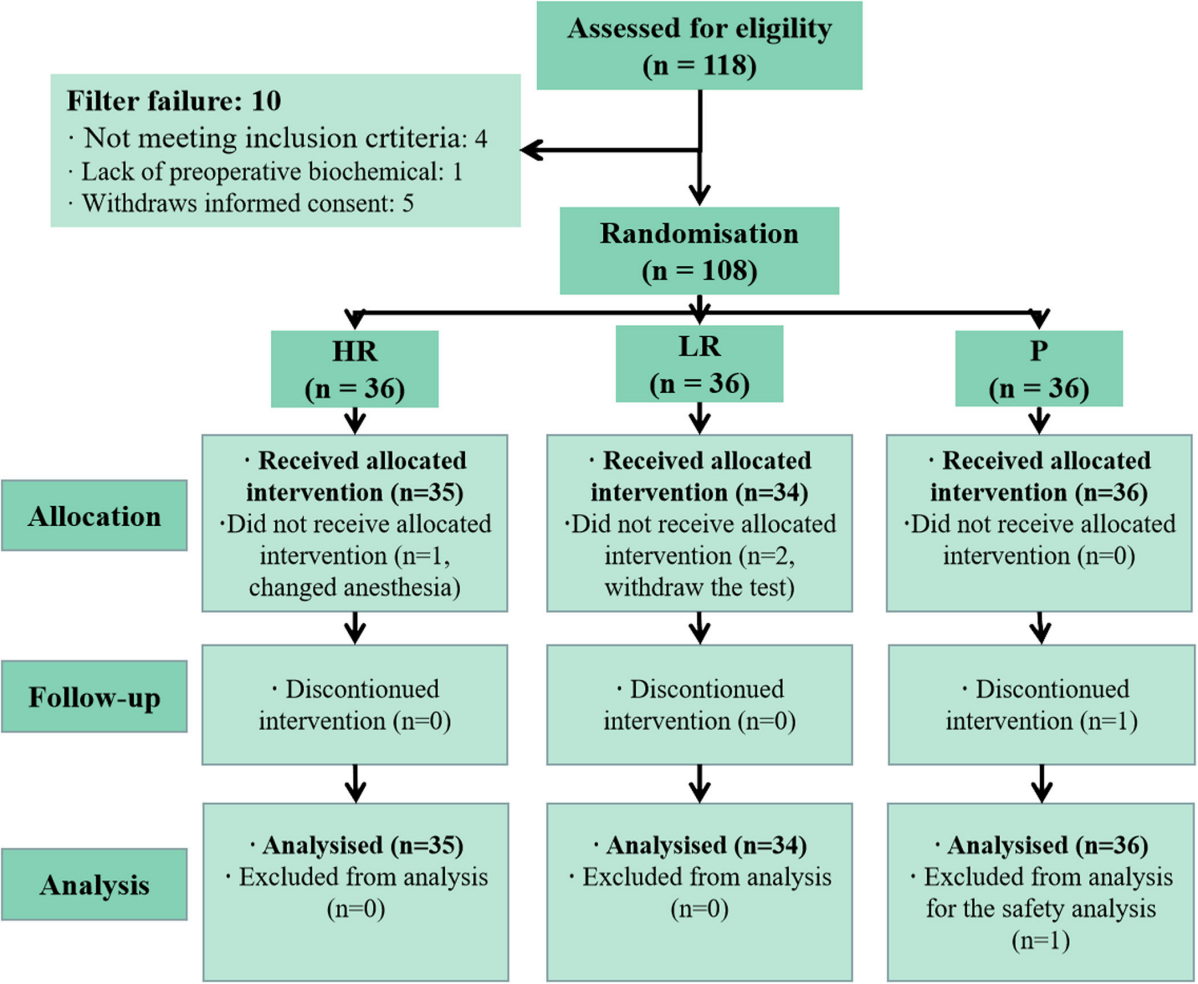
CONSORT flow diagram of the patients' enrollment. CONSORT, Consolidated Standards of Reporting Trials. HR, high‐dosage remimazolam group; LR, /low‐dosage remimazolam group; *n*, number(s); P, propofol group. [Color figure can be viewed at wileyonlinelibrary.com]

From May 6, 2021 to July 4, 2021, a total of 118 patients were selected from 17 hospitals nationwide, in which 108 patients (36 in each group) were randomly enrolled, and 105 patients were used the study medication (Figure 1).

### 3.1 Baseline demographics

There were no statistical differences in the most of demographic (Table 1) and surgical characteristics (Table 2) between the three groups. Intraspinal anesthesia was the most common regional anesthesia methods (71.4%), followed by periphery nerve block (23.8%) and local infiltration anesthesia (4.8%) in this study (Table 2).

**Table 1:** Characteristics of patients.

Characteristics		ALL	HR group	LR group	P group
		*N* = 105	*N* = 35	*N* = 34	*N* = 36
Gender, *n* (%)	Male	56 (53.3)	18 (51.4)	18 (52.9)	20 (55.6)
	Female	49 (46.7)	17 (48.6)	16 (47.1)	16 (44.4)
Age (year‐old)	Mean ± Std	41.0 ± 12.6	36.5 ± 12.6*	42.0 ± 12.5	44.4 ± 11.9
	≧60, *n* (%)	7 (6.7)	1 (2.9)	3 (8.8)	3 (8.3)
Height (cm)	Mean ± Std	165.2 ± 8.4	165.0 ± 8.7	164.6 ± 8.9	166.0 ± 7.6
Weight (Kg)	Mean ± Std	62.3 ± 9.4	62.4 ± 8.6	61.7 ± 10.0	62.7 ± 9.8
BMI (kg/m^2^)	Mean ± Std	22.8 ± 2.6	22.9 ± 2.2	22.7 ± 3.0	22.7 ± 2.6
ASA, *n* (%)	I	62 (59.0)	22 (62.9)	19 (55.9)	21 (58.3)
	II	43 (41.0)	13 (37.1)	15 (44.1)	15 (41.7)
Ethnicity: Han, *n* (%)		100 (95.2)	31 (88.6)	33 (97.1)	36 (100.0)
Previous surgery, *n* (%)		52 (49.5)	18 (51.4)	13 (38.2)	21 (58.3)
Allergy, *n* (%)		6 (5.7)	2 (5.7)	1 (2.9)	3 (8.3)
Hypertension, *n* (%)		8 (7.6)	3 (8.6)	1 (2.9)	4 (11.1)
Diabetes, *n* (%)		4 (3.8)	0 (0.0)	3 (8.8)	1 (2.8)

Abbreviations: ASA, American Society of Anesthesiologists; BMI, body mass index.

* *p* < 0.05, compared with P group.

**Table 2:** Perioperative information.

Characteristics		All	HR group	LR group	P group
		*N* = 105	*N* = 35	*N* = 34	*N* = 36
Type of surgery	Extremities, *n* (%)	62 (59.0)	23 (65.7)	17 (50.0)	22 (61.1)
	Pelvic floor and perineum, *n* (%)	34 (32.4)	11 (31.4)	13 (38.2)	10 (27.8)
	Thoracic and abdominal walls, *n* (%)	9 (8.6)	1 (2.9)	4 (11.8)	4 (11.1)
Types of local anesthesia	Infiltration, *n* (%)	5 (4.8)	0 (0.0)*	4 (11.8)	1 (2.8)
	Nerve block, *n* (%)	25 (23.8)	13 (37.1)*	3 (8.8)	9 (25.0)
	Intraspinal, *n* (%)	75 (71.4)	22 (62.9)*	27 (79.4)	26 (72.2)
Operation time (min)	Mean ± Std	54.2 ± 31.6	60.1 ± 33.3	49.8 ± 32.0	52.7 ± 29.6
Additional analgesics	Once, *n* (%)	4 (3.8)	2 (5.7)	1 (2.9)	1 (2.9)
	Twice, *n* (%)	2 (1.9)	0 (0.0)	1 (2.9)	1 (2.9)
Duration of sedation (min)	Mean ± Std	64.6 ± 36.4	71.6 ± 38.9	61.5 ± 35.2	60.5 ± 34.8
Rescue sedation	*n* (%)	1 (1.0)	0 (0.0)	0 (0.0)	1 (2.9)

Abbreviations: HR, high‐dosage remimazolam group; LR, low‐dosage remimazolam group; P, propofol group; Std, standard deviation.

* *p* < 0.017, Compared with LR group.

### 3.2 Percentage of sedation duration

**Figure 2:**
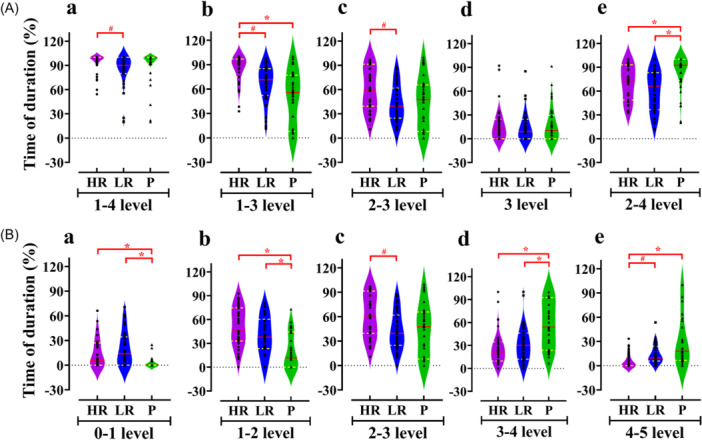
Percentage of minutes of duration at different MOAA/S levels over the entire sedation period. (A) The duration percentage of MOAA/S levels in 1–4 (A‐a), in 1–3 (A‐b), in 2–3 (A‐c), in 3 (A‐d), and in 2–4 (A‐e). (B) The duration percentages between adjacent MOAA/S levels, that is, 0–1 level (B‐a), 1–2 level (B‐b), 2–3 level (B‐c), 3–4 level (B‐d), and 4–5 level (B‐e). HR, high‐dosage remimazolam group; MOAA/S, Modified Observer's Assessment of Alertness/Sedation score; LR, low‐dosage remimazolam group; P, propofol group. *p < 0.05, compared with P group. ^#^ *p* < 0.05, Compared with LR group. [Color figure can be viewed at wileyonlinelibrary.com]

The duration of sedation was 64.6 ± 36.4 min, and only one patient underwent rescue sedation (in group P) among the three groups (Table 2). The duration percentage of MOAA/S levels 1–4 was 100.0 [8.1] %, 89.9 [20.2] %, and 100.0 [7.7] % in the HR, LR, and P groups, respectively (*p*_(HR vs. LR)_ = 0.007) (Figure 2A‐a). The duration percentage of MOAA/S levels 1–3 from high to low was HR, LR, and P groups (*p*_(HR vs. LR)_ < 0.001, *p*_(HR vs. P)_ < 0.001) (Figure 2A‐b). Regarding the duration percentage of MOAA/S levels 2–3, sorted from highest to lowest were HR, P, and LR groups (*p*_(HR vs. LR)_ = 0.009) (Figure 2A‐c). However, the duration percentage of MOAA/S levels 2‐4 was significantly higher in the P group than that in the HR and LR groups (*p*_(HR vs. P)_ = 0.007, *p*_(LR vs P)_ < 0.001) (Figure 2A‐e). At MOAA/S level 3, there was no statistically significant difference in the duration percentages (*p* = 0.853) (Figure 2A‐d).

From the above MOAA/S analysis, it can be found that the HR and LR groups exhibit higher percentages in the lower levels. Then, the duration percentages between adjacent MOAA/S levels were analyzed (Figure 2B). Compared the group P, the duration percentage of MOAA/S levels 0–1 and levels 1–2 was significantly higher in the HR group and LR group (levels 0–1: *p*_(HR vs. P)_ < 0.001, *p*_(LR vs. P)_ < 0.001, Figure 2B‐a; levels 1–2: *p*_(HR vs. P)_ < 0.001, *p*_(LR vs. P)_ = 0.002, Figure 2B‐b), while the duration percentage of MOAA/S levels 3–4 and levels 4–5 was lower (levels 3–4: *p*_(HR vs. P)_ < 0.001, *p*_(LR vs. P)_ = 0.001, Figure 2B‐d; levels 4‐5: *p*_(HR vs. LR)_ < 0.001, *p*_(HR vs. P)_ < 0.001, Figure 2B‐e). And in the duration percentage of MOAA/S levels 2–3, the difference between the HR group and the P group was not statistically significant (Figure 2A‐c and Figure 2B‐c).

**Figure 3:**
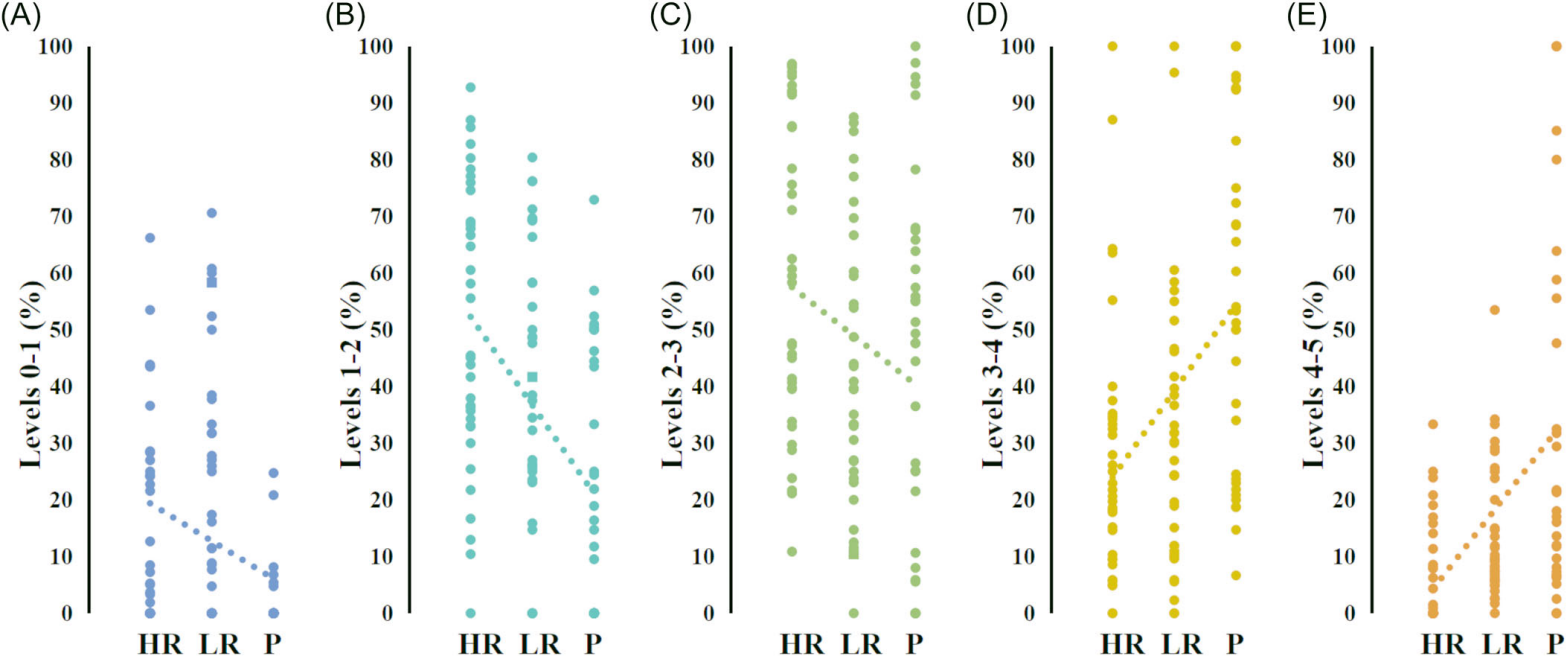
Correlation analysis of the duration percentage of adjacent MOAA/S levels and the groups (HR, LR, and P). By Spearman's rank correlation. (A) Levels 0–1, *p* < 0.001, *r* = −0.366. (B) Levels 1–2, *p* < 0.001, *r* = −0.460. (C) Levels 2–3, *p* = −0.223. (D) Levels 3–4, *p* < 0.001, *r* = 0.411. (E) Levels 4–5, *p* < 0.001, *r* = 0.483). HR, high‐dosage remimazolam group; LR, low‐dosage remimazolam group; MOAA/S, Modified Observer's Assessment of Alertness/Sedation score; P, Propofol group. [Color figure can be viewed at wileyonlinelibrary.com]

In other words, although there was no significant difference in the percentage of target sedation duration (MOAA/S levels 1–4) between the HR or LR group and the P group, the HR or LR group had a significantly higher percentage of deeper sedation (MOAA/S levels 0–2) than the P group. This suggests that remimazolam appears to provide a deeper state of sedation than propofol. Further correlation analysis showed that the duration percentage of adjacent MOAA/S levels, and the groups (HR, LR, and P) were correlated (Figure 3A, levels 0–1: *p* < 0.001, *r* = −0.366. Figure 3B, levels 1–2: *p* < 0.001, *r* = −0.460. Figure 3C, levels 2–3: *p* = −0.223. Figure 3D, levels 3–4: *p* < 0.001, *r* = 0.411. Figure 3E, levels 4–5: *p* < 0.001, *r* = 0.483).

### 3.3 Onset and recovery of sedatives

The percentage of patients in the HR, LR, and P groups who achieved the target sedation (MOAA/S levels 1‐4) within 3 min after administration was 85.7%, 58.8%, and 82.9% (*p*_(HR vs. LR)_ < 0.001), respectively. After drug withdrawal, the overall recovery time from sedation to MOAA/S level 5 was about 7.9 ± 5.7 min with no significant difference between different groups (*p* = 0.971), and the time to Aldrete ≧9 score was about 10.2 ± 5.7 min without statistical difference between three groups (*p* = 0.919) (Table 3).

**Table 3:** Comparison of information on other sedative effects.

Characteristics			ALL	HR group	LR group	P group	*p* value	
			*N* = 105	*N* = 35	*N* = 34	*N* = 36		
Proportion of sedation (MOAA/S) was achieved within 3 min	Level 0	*n* (%)	5 (4.8)	3 (8.6)	0 (0.0)	2 (5.7)	0.001*	HR vs. LR: < 0.001**
	Levels 1–4		79 (76.0)	30 (85.7)	20 (58.8%)	29 (82.9)		HR vs. P: 0.789
	Level 5		20 (19.2)	2 (5.7)	14 (41.2)	4 (11.4)		LR vs. P: 0.005**
Time to return to MOAAS level 5 after discontinuation, min		Mean ± Std	7.9 ± 5.7	7.8 ± 5.1	8.1 ± 5.4	7.8 ± 6.5	0.971	
		Min–Max	0.0–31.0	0.0–21.0	0.0–19.0	0.0–31.0		
Time to Aldrete ≧9 score, min		Mean ± Std	10.2 ± 5.7	10.3 ± 4.7	9.8 ± 4.6	10.3 ± 7.6	0.919	
		Min–Max	1–34	2.0–22.0	1.0–22.0	2–34		
Satisfaction score	Anesthesiologists		9.0 ± 1.5	9.4 ± 1.1	9.4 ± 1.0	8.3 ± 2.1	0.003*	HR vs. LR: 0.997
		Mean ± Std	2–10	5–10	6–10	2–10		HR vs. P: 0.023**
		Min–Max						LR vs. P: 0.030**
	Patient		9.5 ± 1.3	9.7 ± 0.6	9.6 ± 1.1	9.3 ± 1.9	0.306	
			0–10	8–10	5–10	0–10		
Anterograde amnesia		*n* (%)	63 (75.0)	21 (75.0)	24 (80.0)	18 (69.2)	0.650	

Abbreviations: HR, high‐dosage remimazolam group; LR, low‐dosage remimazolam group; MOAA/S, Modified Observer's Assessment of Alertness/Sedation score; P, propofol group.

* *p* < 0.05, the difference among the three groups was statistically significant

** *p* < 0.05, the difference was statistically significant after pairwise comparison.

### 3.4 Satisfaction score

The anesthesiologists' satisfaction was 9.4 ± 1.0 (LR group), 9.4 ± 1.1 (HR group), and 8.3 ± 2.1 (P group) in descending order (*p*_(HR vs. P)_ = 0.023, *p*_(HR vs. LR)_ = 0.030). Patient's satisfaction was approximately 9.5 ± 1.3 (*p* = 0.306) (Table 3). By correlation analysis, anesthesiologist satisfaction was correlated with the duration percentage of MOAA/S levels 1–2 (*p* = 0.039, *r* = 0.204), levels 2–3 (*p* = 0.033, *r* = 0.210), and levels 3‐4 (*p* = 0.011, *r* = −0.248), while patient's satisfaction was only correlated with duration percentage of MOAA/S levels 4‐5 (*p* = 0.033, *r* = −0.209).

### 3.5 Binary logistic regression analysis

Univariate analysis showed that the types of sedatives (vs. HR group, *p*_(LR)_ = 0.042, OR = 2.812, 95% CI: 1.039–7.612; *p*_(P)_ = 0.001, OR = 5.455, 95% CI: 1.960–15.176) and BMI (*p* = 0.039, OR = 0.847, 95% CI: 0.723–0.992) were the influencing factors of shorter duration of appropriate sedation. Including the basic information of patients and perioperative information at the same time, the results showed that the use of sedatives (vs. HR group, *p*_(LR)_ = 0.058; *p*_(p)_ = 0.001, OR = 7.217, 95% CI: 2.147–24.255) and BMI (*p* = 0.027, OR = 0.804, 95% CI: 0.663–0.976) still had an effect on intraoperative sedation.

### 3.6 Anterograde amnesia

Anterograde amnesia occurred in more than half of the patients in the three groups, and the incidence rates were 75% (HR group), 80% (LR group), and 69.2% (P group), with no statistically significant difference (*p* = 0.650) (Table 3).

### 3.7 Safety analysis

Most of the adverse events were either mild or moderate during treatment. There were no adverse events resulting in death or withdrawal from the trial. The main adverse events were injection pain (25%), spontaneous movement during surgery (10.6%), hypertension (14.4%), hypotension (11.5%), bradycardia (30.8%), tachycardia (2.9%), PONV (6.7%), and lower SpO_2_ (5.8%). However, there were no statistical differences in the incidence of adverse events between three groups (Table 4).

**Table 4:** Adverse reactions, *n* (%).

Adverse reactions	All	HR group	LR group	P group
	*N* = 105	*N* = 35	*N* = 34	*N* = 36
Injection pain	26 (25.0)	8 (22.9)	5 (14.7)	13 (37.1)
Spontaneous movement during surgery	11 (10.6)	4 (11.4)	3 (8.8)	4 (11.4)
Hypertension	15 (14.4)	7 (20.0)	4 (11.8)	4 (11.4)
Hypotension	12 (11.5)	3 (8.6)	5 (14.7)	4 (11.4)
Heart rate <50 bpm	32 (30.8)	10 (28.6)	8 (23.5)	14 (40.0)
Heart rate >100 bpm	3 (2.9)	2 (5.7)	0 (0.0)	1 (2.9)
PONV	7 (6.7)	3 (8.6)	1 (2.9)	3 (8.6)
SpO_2_ < 90%	6 (5.8)	2 (5.7)	1 (2.9)	3 (8.6)
Postoperative delirium	0 (0.0)	0 (0.0)	0 (0.0)	0 (0.0)

Abbreviations: HR, high‐dosage remimazolam group; LR, low‐dosage remimazolam group; P, propofol group; bpm, times per minute; PONV, postoperative nausea and vomiting.

## 4 DISCUSSION

In this prospective randomized controlled trial, we found that 0.1 mg/kg BW remimazolam can achieve similar sedation without increasing adverse reactions compared with regional nerve block sedation with 1.0 mg/kg BW propofol and better than 0.05 mg/kg BW remimazolam. However, the proportion of the duration percentage of lower MOAA/S levels (including levels 0–1, 1–2, and 1–3) was significantly higher in the remimazolam group than that in propofol group. Correlation analysis and regression analyses also revealed that remimazolam can provide more depth sedative effects than propofol. Interestingly, doctors are more satisfied by using 0.1 mg/kg BW remimazolam for sedation (vs. 0.05 mg/kg BW remimazolam, vs. 1.0 mg/kg BW propofol), while patient satisfaction did not differ between the three groups. Through regression analysis, the results showed that in addition to the type of sedation had an effect on the duration of sedation, the patient's BMI was also one of the main influencing factors. In summary, remimazolam in 0.1 mg/kg loading can provide a stable and deeper sedation effect for regional anesthesia, with rapid action and no increase in adverse events.

### 4.1 Sedative effect of remimazolam

It is reported that remimazolam was suitable for the induction and maintenance of programmed sedation in adults.^16^ Xiao‐Yan Sheng found that the sedative effect of remimazolam was observed at a dose of 0.05 mg/kg while doses ≥0.075 mg/kg were used to achieve maximum sedation within 1–2 min after injection.^17^ In addition, the study by Worthington et al. in colonoscopy showed that the median MOAA/S achieved within 5 min by remimazolam with an induction dose of 0.1 mg/kg was shorter than that by remimazolam with 0.04 mg/kg.^18^ These results help us to explain the faster onset of action of remimazolam at an induction dose of 0.1 mg/kg compared with 0.05 mg/kg. It should be noted that the above studies all showed that remimazolam has a high clearance rate and short half‐life. Kilpatrick et al. also confirmed that the elimination half‐life of remimazolam did not change significantly with the prolongation of administration time,^19^ which also explains why there is no significant difference in recovery time between the two remimazolam groups. Therefore, 0.1 mg/kg remimazolam can provide better sedation than 0.05 mg/kg remimazolam in patients undergoing regional anesthesia.

The duration percentage of MOAA/S of levels 1–4, the percentage of patients reaching the target sedation within 3 min, and the recovery time after drug withdrawal did not differ significantly between the HR and P groups. Chen applied remimazolam in endoscopy and found that compared with propofol, the induced dose of 5 mg (about 0.05‐0.1 mg/kg) of remimazolam could achieve sedative effect similar to that of propofol without affecting the patient's recovery.^20^ Similar finding was found in a study of hysteroscopy.^21^ Therefore, the sedative effect of remimazolam 0.1 mg/kg during regional anesthesia was comparable to that of propofol in this study, consistent with the results of previous studies related to sedation.

In the remimazolam group, the proportion of the duration percentage of lower MOAA/S levels (including levels 0–1, 1–2, and 1–3) was significantly higher than that in the propofol group. Correlation analysis and regression analyses also revealed that remimazolam can provide more depth sedative effects than propofol. Shao‐hui Chen et al. used remimazolam in 381 upper gastrointestinal endoscopy examinations and found that 5.0 mg remimazolam had significantly longer duration of MOAA/S ≤ 3 than propofol.^21^ In addition, the higher the dose of remimazolam, the higher the percentage of duration of low MOAA/S levels. In a phase I study of 60 healthy Chinese volunteers, the results showed that the MOAA/S rating decreased as the dose of remimazolam increased.^17^ Moreover, the previous literature^18^, ^19^ has shown that higher doses of remimazolam can provide deeper sedative effects.

### 4.2 The satisfaction of anesthesiologists and patients

In this study, it was clear that the sedative effect of 0.1 mg/kg loading dose of remimazolam was significantly better than that of 0.05 mg/kg of remimazolam and comparable to 1.0 mg/kg of propofol. However, the satisfaction with sedation varied between anesthesiologists and patients. Anesthesiologists considered that remimazolam at loading doses of 0.05 and 0.1 mg/kg was both superior to propofol for sedation. But there was no significant difference in patients' satisfaction among the three groups. Through the correlation analysis, we found that anesthesiologists seem to prefer deeper sedation (MOAA/S levels 1–3), while patient's satisfaction with sedation was only inversely correlated with the duration percentage of MOAA/S levels 4–5. In other words, doctors prefer a deeper degree of sedation, while the patient only cares about reaching a state of sedation. In a deeper state of sedation, patients will not be disturbed by strong stimuli, such as surgical traction. This may be the reason for the difference of sedation satisfaction between the anesthesiologists and patients. A prospective randomised controlled study of 72 patients undergoing endoscopic submucosal dissection showed that a stable and satisfactory procedure in the eyes of the endoscopist does not necessarily translate into patient satisfaction. But they did not further analyze the correlation between MOAA/S levels and patient satisfaction. Interestingly, after giving midazolam in advance, the authors found that the satisfaction of patients was consistent with that of endoscopists.^22^ The additional use of midazolam will undoubtedly increase sedation, which make more patients reach the ideal sedation state.

### 4.3 The BMI of patients may influence sedation state

A low BMI is often associated with undernourishment,^23^ indicating potential lower levels of plasma proteins, which can influence the effectiveness of sedative drugs. Available studies suggest that 25% of the proteins in the human proteome circulate in the blood, with many drug targets being among these proteins.^24^ And Goudswaard LJ's work has demonstrated that BMI has a systemic effect on the circulating proteome.^25^ These studies explain why the BMI may have an impact on the sedation effect of patients.

### 4.4 Safety of remimazolam

Compared with the loading dose of 1.0 mg/kg propofol, the loading dose of 0.05–0.1 mg/kg remimazolam does not increase the adverse events. And the most common adverse events were bradycardia, injection pain, and blood pressure fluctuation. Most of the relevant literature showed that compared with propofol, remimazolam can significantly reduce the adverse events of patients, and the incidence of bradycardia, hypotension, and respiratory depression was lower.^20^, ^21^ However, the induction of propofol in these studies was 1.5–2.0 mg/kg, which was higher than the induction used in our study (1.0 mg/kg).

### 4.5 Both remimazolam and propofol have a higher anterograde amnesia effect

In this study, the incidence of anterograde amnesia was about 69.2%–80.0%, whether remimazolam or propofol. Research has been reported that as remimazolam is a benzodiazepine, it may cause anterograde amnesia (similar to other benzodiazepines).^26^ Propofol‐induced anterograde amnesia has been previously reported, and the literature showed that its anterograde amnesia was dose dependent.^27^ However, there was no study comparing the incidence of anterograde amnesia between these two sedatives.

### 4.6 Strengths and limitations

This study was the first to investigate the efficacy, safety, and appropriate dose of remimazolam for regional anesthesia sedation. Compared with propofol (1.0 mg/kg), 0.1 mg/kg remimazolam demonstrated comparable onset and offset rates, as well as duration of sedation. Moreover, remimazolam induced a deeper level of sedation, accompanied by a degree of anterograde amnesia, while not prolonging recovery time or increasing the incidence of adverse events. Unfortunately, due to the relatively small sample size, further large‐scale studies are warranted to provide additional insights and confirm these findings.

## 5 CONCLUSION

Remimazolam (0.1 mg/kg loading dose followed by a maintenance infusion of 0–3 mg/kg/h) can provide a stable and deeper sedation effect for regional anesthesia, and the effect was rapid, with a certain anterograde amnesia, no increase in adverse events, which can further expand its clinical application.

## AUTHOR CONTRIBUTIONS

Fei Liu was involved in the overall design and conception; Ting‐Ting Li, Yue‐Xin Huang, and Genoveva Barros da Graca Cunha were responsible to the statistics collection and analysis; Fei Liu, Lu Yin, and Yong Wang conducted and reported the operations; Ting‐Ting Li, Xiu‐Hong Wang, and Shi‐Wei Yang were responsible for drafting the manuscript; Yan‐Huan Wei participated in draft writing and polishing. All authors have read and approved the final version of the manuscript.

## CONFLICTS OF INTEREST STATEMENT

Fei Liu is the editorial member of Ibrain and a coauthor of this article. She was excluded from editorial decision‐making related to the acceptance and publication of this article. Editorial decision‐making was handled independently by Editor‐in‐Chiefs to minimize bias. The remain authors declare that there are no competing interests.

## ETHICS STATEMENT

This study was conducted in accordance with Good Clinical Practice Guidelines and the principles of the Declaration of Helsinki, approved by the local ethics committee (Ethics Committee on Clinical Trial, West China Hospital of Sichuan University, NO. HX‐IRB‐AF‐14‐V4.0, Clinical trial (Western medicine) Review No. 35, 2021.) and Clinical trial by State Drug Administration (No. CXHL2000187). This study has been registered with the Chinese Clinical Trial Registry (No. ChiCTR2100054956). Written informed consent was obtained from all subjects, a legal surrogate, and the parents or legal guardians for minor subjects, or the requirement for written informed consent was waived by the IRB. All the information of CONSORT 2010 checklist was followed in this study.

## Supporting information

Supporting information.

## Data Availability

All data generated during this study are included in this published article and its supplementary information files. Further minor datasets are available from the corresponding author on reasonable request.
