# Supplementary material for: Efficacy and safety of remimazolam versus propofol for intraoperative sedation during regional anesthesia: A phase II, multicenter, randomized, active‐controlled, single‐blind clinical trial
Source: Ibrain. 2024 Jun 16;10(2):134–45. doi: 10.1002/ibra.12163 (PMC11193864; doi:10.1002/ibra.12163)
Supplement: Supplementary file 1 — Supporting information. [file IBRA-10-134-s001.docx]

**Supplementary Document**

**Table 1. Information of each research center and the inclusion of cases**

| **NO.** | **Name of Research Centre** | **Group** | | | **In all** |
| --- | --- | --- | --- | --- | --- |
|  |  | **HR** | **LR** | **P** |  |
| 01 | West China Hospital of Sichuan University, Sichuan | 0 | 1 | 3 | 4 |
| 02 | Sichuan Provincial People's Hospital, Sichuan | 2 | 2 | 1 | 5 |
| 03 | The Second People's Hospital of Nanning, Guangxi | 6 | 3 | 1 | 10 |
| 04 | Affiliated Hospital of Guizhou Medical University, Guizhou | 0 | 2 | 0 | 2 |
| 07 | Union Hospital Affiliated to Tongji Medical College, Huazhong University of Science and Technology, Wuhan | 2 | 0 | 3 | 5 |
| 08 | The Second Affiliated Hospital of Nanchang University, Jiangxi | 0 | 2 | 1 | 3 |
| 10 | The Second Affiliated Hospital of Wenzhou Medical University, Zhejiang | 6 | 2 | 2 | 10 |
| 11 | Huzhou Central Hospital, Zhejiang | 2 | 1 | 3 | 6 |
| 12 | Guangzhou First People's Hospital, Guangdong | 0 | 1 | 0 | 1 |
| 13 | Dongguan People's Hospital, Guangdong | 1 | 6 | 1 | 8 |
| 14 | Zhangzhou Hospital, Fujian | 4 | 4 | 2 | 10 |
| 15 | Jinan Central Hospital, Shandong | 4 | 2 | 6 | 12 |
| 16 | The Second Affiliated Hospital of Anhui Medical University, Anhui | 4 | 3 | 6 | 13 |
| 17 | Nanjing First Hospital, Jiangsu | 1 | 2 | 1 | 4 |
| 19 | Changzhi People's Hospital, Shanxi | 1 | 0 | 1 | 2 |
| 20 | Henan Provincial People's Hospital, Henan | 2 | 3 | 1 | 6 |
| 23 | Meihekou Central Hospital, Jilin | 0 | 0 | 4 | 4 |
| **In all** | | 35 | 34 | 36 | 105 |

Note: No. = Number. HR = 0.1 mg/kg Remimazolam loading dose group. LR = 0.05 mg/kg Remimazolam loading dose group. P = 1.0 mg/kg Propofol loading dose group.
